# Infectious crystalline keratopathy associated with *Klebsiella oxytoca*

**DOI:** 10.1007/s12348-012-0071-0

**Published:** 2012-03-25

**Authors:** Timothy Y. Chou, Rohit Adyanthaya

**Affiliations:** State University of New York, HSC L2, Room 152, Stony Brook, NY 11794 USA

**Keywords:** Infectious crystalline keratopathy, ICK, *Klebsiella oxytoca*, *Staphylococcus*, Keratitis

## Abstract

**Purpose:**

The purpose of this study is to report a novel case of a *Klebsiella oxytoca-associated* infectious crystalline keratopathy

**Methods:**

This is a case report study.

**Results:**

An 80-year-old woman presented with complaint of noticing a white spot in the left eye for 2 to 3 days, as well as mild soreness and discharge. Past ocular history was notable for a failed left corneal transplant for which she was taking prednisolone acetate 1 % twice per day. On slit-lamp examination, there was an extensive stromal ulcer and infiltrate in the inferior half of the transplant. Extending superiorly in the graft were branching, needle-like deep stromal opacities, characteristic of infectious crystalline keratopathy. Diagnostic scrapings revealed Gram-negative bacilli, subsequently identified on culture as *K. oxytoca*. There was also light growth of *Staphylococcus* species. The patient was placed on double topical antibiotic therapy with moxifloxacin and fortified tobramycin. After 2 months of treatment there was gradual resolution of the infection.

**Conclusions:**

*K. oxytoca* is a microorganism which can be associated with clinical infectious crystalline keratopathy, presenting as a mixed infection along with *Staphylococcus* species.

## Introduction

Infectious crystalline keratopathy (ick) is a slowly progressing corneal infection characterized by branching, grayish-white, needle-like opacities within the corneal stroma, with a paucity of corneal and anterior segment inflammation [1, 2]. The crystalline deposits are composed of bacteria aggregating and spreading within the corneal stromal lamellae. The bacteria are enveloped in a biofilm composed of an exopolysaccharide glycocalyx. This biofilm isolates the infectious organisms from the immune system, as well as conferring resistance to antimicrobial penetration [3]. Risk factors for infectious crystalline keratopathy include previous corneal surgery, long-term topical corticosteroid use, prior corneal disease, and systemic immunocompromise [2]. It has been reported after cataract extraction, penetrating keratoplasty, corneal refractive surgery, and glaucoma filtering surgery. Many microbial pathogens have been reported to cause infectious crystalline keratopathy (ICK), especially Gram-positive cocci, and streptococci in particular [4]. In this report, we present the first case of a patient with infectious crystalline keratopathy associated with *Klebsiella oxytoca*.

## Case report

An 80-year-old woman presented with complaint of noticing a white spot in her left eye for 2 to 3 days. There was mild soreness and discharge. The patient had a complicated past ocular history involving that eye, beginning with significant myopic degeneration. Nineteen years prior, she had undergone left eye cataract surgery. Eleven years later she sustained a ruptured left globe. Despite emergent repair, she developed corneal blood staining and corneal decompensation, requiring penetrating keratoplasty. The graft ultimately failed, and was not repeated due to limited visual potential. At the time of presentation, she was being maintained on prednisolone acetate 1 %, twice per day.

**Fig. 1:**
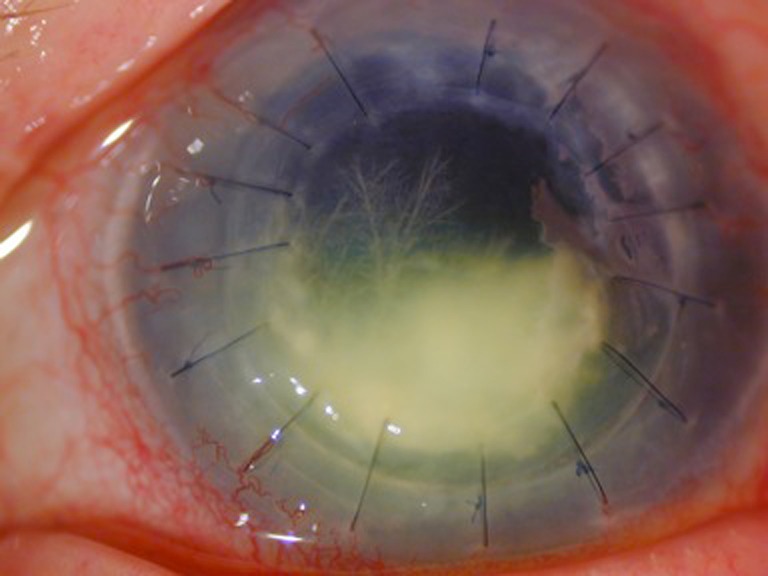
Slit-lamp photograph of infectious infiltrate in the graft. There are branching needle-like stromal opacities extending upwards characteristic of infectious crystalline keratopathy

On examination, visual acuity was hand motion in the left eye. Slit-lamp examination revealed minimal conjunctival injection. There was an extensive stromal infiltrate in the transplant involving much of its inferior and central aspects, bordered by the graft edge. Extending upward into the superonasal graft were deep branching, needle-like stromal opacities (Fig. 1).

Based on the clinical picture, the patient was diagnosed with an infectious keratitis, manifesting as infectious crystalline keratopathy. diagnostic corneal scrapings were performed; sutures within the infiltrate were removed and cultured as well. Gram staining revealed Gram-negative bacilli. The organism was identified as *K. oxytoca* on culture. There was also a minor growth of *Staphylococcus* species. The patient was placed on intensive topical antibiotic therapy utilizing moxifloxacin 0.3 % and fortified tobramycin 15 mg/ml. After 2 months of treatment, there was gradual resolution of the infection, with healing of the ulcer and scarring-in of the infiltrate.

## Discussion

Diagnosis of ICK is primarily established by clinical appearance on slit-lamp examination. Isolation of the infectious organism can often be difficult since the infiltrate may often lie deep within the corneal stroma, inaccessible to superficial scrapings. Needling of the crystals, culturing involved sutures, or corneal biopsy may be required [5]. At times, a specific pathogen has only been identified after therapeutic corneal transplantation [6]. Polymerase chain reaction has also been demonstrated to be a useful diagnostic tool [7].

Many microorganisms have been identified as causative agents for ICK. Gram-positive alpha-hemolytic streptococci, typically of the Viridans group, are the most common pathogens encountered [2, 4]. Additional Gram-positive bacteria that have been isolated include enterococci, *Staphylococcus aureus*, and other staphylococcal species, *Gemella haemolysans*, and anaerobes such as *proprionobacterium acne* [2, 4, 5]. Non-bacterial organisms have also been implicated in ICK, like filamentous fungi and yeasts including *Candida* [2, 4, 8], and in mixed infections associated with *Acanthamoeba* [2, 9]. A few case reports have highlighted the occurrence of ICK after LASIK surgery, caused by microbes such as nontuberculous mycobacteria [10] and *Alternaria* species [8]. Gram-negative bacteria likewise have not escaped notice, with *Serratia marcescens*, various species of *Pseudomonas* and *Hemophilus*, and even the spirochete *Borrelia garinii* having been described [2, 4, 6, 7, 11, 12].

To the best of our knowledge, this is the first actual clinical report of infectious crystalline keratopathy associated with *K. oxytoca*. A typical Gram-negative keratitis of this size would usually present acutely with severe pain, deep injection, purulent discharge, keratolysis, and hypopyon. We believe that the patient's subacute and pauci-inflammatory clinical presentation indicates that the klebsiella was a true and integral contributor to the ICK infection itself. *K. oxytoca* is a Gram-negative rod, in the enterobacteriaceae family. It is a common cause of antibiotic-associated, *Clostridium difficile*-negative, hemorrhagic colitis in immunocompetent individuals [13]. It is also an infectious agent involved in meningitis, adrenal hemorrhage and septic shock in immunocompromised patients. An in vitro model of ICK has been created by inoculating *K. oxytoca* onto corneal buttons [14].

The exact role of the *Staphylococcus* species that was cultured concomitantly in our patient is unclear. It was thought to be a possible contaminant because of lighter growth, but that it may have been a co-infectious agent is also possible. We do know that there have been several descriptions of polymicrobial ICK [4, 9, 11]. In these instances, one of the isolates may be considered as the primary [4]. We would propose that the infectious milieu of a polymicrobial keratitis is one of those settings that can promote ICK. Tu and colleagues touch on this concept with their suggestion of possible “endosymbiosis” between co-infectious agents, in their report of acanthamoeba keratitis complicated by concomitant ICK from different Gram-positive cocci [9].

Medical treatment of ICK is often difficult, and involves extended use of antibiotics targeted against the etiologic organism. The Nd:YAG laser, intrastomal antibiotic injection, and even the act of corneal biopsy have been found to be useful adjunctive therapy, presumably in part by disrupting the microbial biofilm [3, 5, 15]. Therapeutic penetrating keratoplasty may be required in up to 50 % of cases [2]. Double antibiotic therapy targeted toward the cultured pathogen was effective in resolving our patient's infection. More study may be required to determine if this approach results in faster resolution than monotherapy, or reduces the need for surgical intervention.

## Conclusions

*K. oxytoca* is a microorganism that can be associated with infectious crystalline keratopathy. It may be present in the form of a mixed microbial keratitis along with *Staphylococcus* species. Treatment may be prolonged, but double topical antimicrobial therapy can be effective in eradicating the infection.
